# Constitutive Negative Regulation of R Proteins in *Arabidopsis* also via Autophagy Related Pathway?

**DOI:** 10.3389/fpls.2016.00260

**Published:** 2016-03-04

**Authors:** Tamara Pečenková, Peter Sabol, Ivan Kulich, Jitka Ortmannová, Viktor Žárský

**Affiliations:** ^1^Laboratory of Cell Biology, Institute of Experimental Botany, Academy of Sciences of Czech RepublicPrague, Czech Republic; ^2^Laboratory of Cell Morphogenesis, Department of Experimental Plant Biology, Faculty of Science, Charles University in PraguePrague, Czech Republic

**Keywords:** resistance, autophagy, R, Avr, ETI, dwarf, lesions, exocyst

## Abstract

Even though resistance (R) genes are among the most studied components of the plant immunity, there remain still a lot of aspects to be explained about the regulation of their function. Many gain-of-function mutants of R genes and loss-of-function of their regulators often demonstrate up-regulated defense responses in combination with dwarf stature and/or spontaneous leaf lesions formation. For most of these mutants, phenotypes are a consequence of an ectopic activation of R genes. Based on the compilation and comparison of published results in this field, we have concluded that the constitutively activated defense phenotypes recurrently arise by disruption of tight, constitutive and multilevel negative control of some of R proteins that might involve also their targeting to the autophagy pathway. This mode of R protein regulation is supported also by protein–protein interactions listed in available databases, as well as *in silico* search for autophagy machinery interacting motifs. The suggested model could resolve some explanatory discrepancies found in the studies of the immunity responses of autophagy mutants.

## Introduction

There are several approaches how to study and classify the plant immunity related events, and the most widespread is division of the plant immunity into two modes – a pathogen-associated molecular patterns (PAMPs) triggered immunity (PTI), which is triggered usually by recognition of structural components of pathogen on the surface of the host cell, and effector triggered immunity (ETI; Jones and Dangl, 2006). These two defense modes employ basically the same means, but PTI is more general and mild, while ETI is much stronger and more efficient. ETI is triggered by the direct or indirect interaction between a specific disease resistance (R) protein and a corresponding avirulence (Avr) protein of pathogen and is accompanied by a number of changes within the plant – production of reactive oxygen species (ROS) by an oxidative burst, accumulation of the salicylic acid (SA), and the transcriptional activation of genes involved in defense response, that lead to a possible final stage – localized programmed cell death called the hypersensitive response (HR; Pontier et al., 1998; review in McDowell and Woffenden, 2003; Vlot et al., 2008).

Disease resistance (R) genes are central components of the plant immune response. All R proteins contain at least some of basic motifs – either Toll/interleukin-1 receptor (TIR) or coiled-coil (CC) structure on the N terminal part, nucleotide-binding site (NBS), leucine-rich repeat LRR), protein kinase and transmembrane domains (review by Martin, 1999; Liu et al., 2007). There are 145 putative genes encoding a product with a TIR domain and 51 with CC domain predicted in the *Arabidopsis thaliana* Col-0 genome (Meyers et al., 2003; Jacob et al., 2013). Majority encode proteins with TIR, NBS, and LRR domains, making the TNL group; some genes encode proteins with TIR and NBS domains but no LRR domain (TN genes) and some encode proteins with a TIR domain only (TX genes; Meyers et al., 2003; Nandety et al., 2013). Besides CC-NBS-LRR containing proteins which make CNL group, there are also four proteins that have NBS motifs similar to CNLs, but lack a CC motif (Meyers et al., 2003).

There are several important molecules involved in signaling downstream the successful R-Avr recognition – ENHANCED DISEASE SENSITIVITY 1 (EDS1), PHYTOALEXIN DEFICIENT 4 (PAD4), NON-RACE SPECIFIC DISEASE RESISTANCE 1 (NDR1) and SENESCENCE ASSOCIATED GENE 101 (SAG101), which are essential for the accomplishment of HR and for the accumulation of the SA. EDS1, PAD4 and SAG101 are involved in transferring signals mainly from TNL proteins, while CNL pathway mostly relies on signaling through NDR1 (Century et al., 1997; Feys et al., 2001; He and Gan, 2002; Wagner et al., 2013).

In *Arabidopsis* mutants in genes coding for R and R-associated proteins, along with defense related deviations, two other most frequent phenotypes are a dwarf stature and a spontaneous HR lesion formation; many times present even simultaneously (**Table 1**). Rarely, a lethal phenotype occurs as well, even though no developmental function for these genes has been found so far. We could notice that for most of the R genes mutants, described phenotypes are a consequence of their activation, in some cases even a gain of function mutations (GOF). Based on the comparison of different studies of plant immunity, our hypothesis aims to suggest a model in which the hyper immune phenotypes arise as a result of disruption of tight, multistep and constitutive negative control of R proteins that possibly involves also their inactivation by the autophagy pathway.

**Table 1:** List of *Arabidopsis* mutants related to R proteins hyper activity causing dwarf and lesion mimic phenotypes.

Gene	Name	Function category	Related mutant phenotypes	Reference
*acd11*	*accelerated cell death 11*	Sphingosine transfer protein	Lesions	Brodersen et al., 2002
*adr1*	*activated disease resistance 1*	CNL	Lesions suppression, dwarf oe	Grant et al., 2003
*adr1-l1*	*activated disease resistance 1-like 1*	CNL	Lesions suppression, dwarf oe	Collier et al., 2011
*adr1-l2*	*activated disease resistance 1-like 2*	CNL	Lesions suppression	Bonardi et al., 2011
*atg5*	*autophagy related gene 5*	Autophagy, ubiquitin ligase	Early senescence	Thompson et al., 2005
*atg6*	*autophagy related gene 6/Beclin1*	Autophagy activation	Pollen-lethality	Fujiki et al., 2007
*atg7*	*autophagy related gene 7*	Autophagy, ubiquitin activating enzyme	Defense-related	Doelling et al., 2002
*atg8*	*autophagy related gene 8*	Ubiquitin-like protein, cargo recruitment	/	Ketelaar et al., 2004
*bak1*	*brassinosteroid-insensitive associated 1*	Receptor-like protein kinase	Semidwarf	Li et al., 2002
*bap1*	*bon1-associated protein*	Calcium-dependent phospholipid-binding	/	Hua et al., 2001
*bir1*	*bak1-interacting receptor-like kinase 1*	Receptor-like protein kinase	Dwarf	Gao et al., 2009
*bon1*	*bonzai1*	Copine-like, membrane trafficking	Dwarf	Hua et al., 2001
*bon2*	*bonzai2*	Copine-like, membrane trafficking	Dwarf	Yang et al., 2006
*bon3*	*bonzai3*	Copine-like, membrane trafficking	Dwarf	Yang et al., 2006
*chs2*	*chilling-sensitive 2*	TNL	Lesions	Huang et al., 2010
*chs3*	*chilling-sensitive 3*	TNL	Lesions	Yang et al., 2010
*cpr1*	*constitutive expresser of pathogenesis-related gene*	F-box protein	Dwarf	Gou et al., 2012
*dnd1*	*defense no death 1*	Cyclic nucleotide-gated ion channel	Dwarf	Yu et al., 1998
*eds1*	*enhanced disease sensitivity 1*	R related signaling	Lesions suppression	Rogers and Ausubel, 1997
*exo70A1*	*exo70A1*	Membrane trafficking	Dwarf	Synek et al., 2006
*exo70B1*	*exo70B1*	Membrane trafficking	Lesions	Kulich et al., 2013
*fls2*	*flagellin*-*sensitive 2*	Receptor-like protein kinase	Defense related	Gomez-Gomez and Boller, 2000
*laz4*	*lazarus 4*	Membrane trafficking	Lesion suppression	Munch et al., 2015
*laz5*	*lazarus 5*	R protein	Lesions suppression	Palma et al., 2010
*lsd1*	*lesion simulating disease 1*	Cell death related	Lesions	Kliebenstein et al., 1999
*mkk1/mkk2*	*mitogen-activated protein kinase kinase kinase 1/2*	Signaling	Dwarf, lesions	Qiu et al., 2008
*mpk1*	*mitogen-activated protein kinase 1*	Signaling	Dwarf	Bartels et al., 2009
*mpk4*	*mitogen-activated protein kinase 4*	Signaling	Dwarf, lesions	Petersen et al., 2000
*ndr1*	*non-race specific disease resistance 1*	R related signaling	Lesions suppression	Century et al., 1995
*pad4*	*phytoalexin deficient 4*	R related signaling	Lesions suppression	Jirage et al., 1999
*rar1*	*required for mlo12 resistance 1*	R related signaling	Lesions suppression	Azevedo et al., 2002
*rin4*	*rpm1-interacting protein 4*	Immunity related	Embryo lethal	Mackey et al., 2002
*rpm1*	*resistance to p. syringae pv maculicola 1*	R protein	Defense related	Debener et al., 1991
*rps2*	*pesistant to p. syringae 2*	R protein	Defene related	Yu et al., 1993
*sag101*	*senescence associated gene 101*	R related signaling	/	Feys et al., 2005
*sgt1b*	*suppressor of g-two allele of skp1*	R related signaling	Lesions suppression	Azevedo et al., 2002
*sid2*	*salicylic acid insensitive 2*	SA synthesis	Defense related	Nawrath and Metraux, 1999
*slh1*	*sensitive to low humidity 1*	R protein	Lesions	Noutoshi et al., 2005
*snc1*	*suppressor of npr1 constitutive 1*	R protein	Dwarfism suppression	Li et al., 2010
*srfr1*	*suppressor of rps4-rld 1*	Tetratricopeptide repeat domain containing	Dwarf	Kim et al., 2010
*ssi2*	*suppressor of SA insensitivity of npr1-5 2*	Stearoyl-ACP desaturase	Dwarf, lesions	Sekine et al., 2004
*ssi4*	*Suppressor of SA insensitivity of npr1-5 4*	R protein	Dwarf	Shirano et al., 2002
*summ2*	*Suppressor of mkk1 mkk2 2*	R protein	Dwarfism and lesions suppression	Zhang et al., 2012
*syp121/syp122*	*Syntaxin 121/syntaxin 122*	Membrane trafficking	Dwarf, lesions	Zhang et al., 2008
*syp23*	*Syntaxin 23*	Membrane trafficking	Semi-dwarf	Ohtomo et al., 2005
*syp31*	*Syntaxin 31*	Membrane trafficking	/	Chatre et al., 2009
*TN2*	*TIR-NBS 2*	R protein	Lesions suppression	Zhao et al., 2015
*TX At2g32140*	*/*	R protein	Dwarf	Kato et al., 2014

## Of Dwarfs and Lesions

It was shown that mutants with over activated R protein dependent defense response develop mostly two phenotypes – dwarfism and/or necrotic leaf lesions (reviewed e.g., in Lorrain et al., 2003 and Janda and Ruelland, 2014). For instance, in plants overexpressing a CNL gene *ACTIVATED DISEASE RESISTANCE 1* (*ADR1*), a constitutive defense response and a dwarf phenotype were found (Grant et al., 2003). A TNL protein SUPPRESSOR OF NPR1 CONSTITUTIVE 1 (SNC1) was found to be overactive in the *bonzai1-1* (*bon1-1*) mutant which also shows a constitutive defense response and reduced plant size (Yang and Hua, 2004). Along with *bon1*, several other autoimmune dwarf mutations were found to be suppressed by mutation of SNC1 locus; namely in *BON1-ASSOCIATED PROTEIN* (*bap1*), *BAK1-INTERACTING RECEPTOR-LIKE KINASE 1* (*bir1*), *SUPPRESSOR OF RPS4-RLD 1* (*srfr1*), *CONSTITUTIVE EXPRESSER OF PATHOGENESIS-RELATED GENE* (*cpr1*) and *MITOGEN-ACTIVATED PROTEIN KINASE 1* (*mpk1*; review in Gou and Hua, 2012). Plants overexpressing a TIR-X gene At2g32140 show also dwarf phenotype and activated expression of defense-related genes (Kato et al., 2014). This phenotype was dependent on EDS1, PAD4, and partially dependent on SALICYLIC ACID INDUCTION DEFICIENT 2 (SID2).

HR-like spontaneous leaf necrotic lesions were found to be even more frequently associated with the mutations in R genes and constitutively activated immunity. For instance, a GOF mutant in TNL RPP4 locus called *chilling sensitive 2* (*chs2*) shows lesions in the low temperature conditions (Huang et al., 2010). GOF *Arabidopsis* mutant in the other *CHS* gene, *chs3-1*, which encodes an unconventional disease resistance (R) protein belonging to the TIR-NB-LRR class with a zinc-binding LIM domain (Lin-11, Isl-1 and Mec-3 domains) at the carboxyl terminus, shows arrested growth, chlorosis and constitutively activated defence responses at 16°C (Yang et al., 2010). A mutant in TNL gene *ssi4* develops chlorotic lesions which can be suppressed by high humidity (Shirano et al., 2002; Zhou et al., 2004). In addition, there are several examples of mutants with spontaneous lesions induction which are suppressed by mutations in loci encoding R proteins of CNL type – *ACTIVATED DISEASE RESISTANCE 1* – *adr1, adr1-l1* and *adr1-l2* suppress *LESION SIMULATING DISEASE 1* (*lsd1*) by down regulating SA signaling (Bonardi et al., 2011; Roberts et al., 2013). Likewise, when a putative TNL encoded by *LAZARUS 5* (*LAZ5*) gene is mutated, *accelerated cell death 11* (*acd11*) lesion phenotype can be suppressed (Palma et al., 2010). It was also shown, that in the absence of the copine-like proteins BON1 and BON3 function, several R-like genes of the TNL/TN type were found to trigger lesion cell death (LCD; Li et al., 2009). Mutation in *SUPPRESSOR OF MKK1 MKK2 2* (*summ2*) which encodes putative NB-LRR, suppresses lesions formation and dwarfism of mutants of MAP kinase pathway *mkk1/mkk2 and mpk4* (Kong et al., 2012).

There are genes coding for other defense related components that when mutated trigger the same constitutive immunity activation and dwarf or/and lesion mimic phenotypes – e.g., *CONSTITUTIVE EXPRESSOR OF PATHOGENESIS-RELATED GENE 1* (*CPR1*), *SUPPRESSOR OF SALICYLIC ACID INSENSITIVITY OF NPR1-5* 2 (*SSI2*), *DEFENSE NO DEATH* 1 (*DND1*), TYPE III *PHOSPHATIDYLINOSITOL-4-KINASES β1β2* (*PI4KIII*β1β2) (Bowling et al., 1994; Yu et al., 1998; Zhang et al., 2003; Sekine et al., 2004; Gou et al., 2012; Sasek et al., 2014). As a regular aspect of these mutants’ phenotype deviations, hyper accumulation of SA was observed.

## Lethality of the Hub

Overactive immunity can disturb plant growth and fitness, and in an extreme case, this can be deleterious. Unexpectedly, an embryo lethal phenotype was found for LOF mutation of a defense related gene *RPM1-INTERACTING PROTEIN 4* (*RIN4*). Being evolutionarily conserved protein in plants, RIN4 is targeted to the plasma membrane by C-terminal acylation, and is required for the activation of a CNL *RESISTANCE TO PSEUDOMONAS SYRINGAE PV. MACULICOLA 1* (RPM1; Kim et al., 2005; Takemoto and Jones, 2005). RIN4 is phosphorylated upon infection with *P. syringae* expressing either AvrB or AvrRpm1 (Mackey et al., 2002). RIN4 is also involved in the activation of another CNL type R protein *RESISTANCE TO P. SYRINGAE 2* (RPS2) by putative Cys protease AvrRpt2 of *P. syringae*, which causes posttranscriptional cleavage and disappearance of RIN4 and this is required for full RPS2 activation (Axtell and Staskawicz, 2003; Mackey et al., 2003). Interestingly, in coimmunoprecipitation experiments, RIN4 was found to associate with RPM1, RPS2 as well as with pathogen recognition receptor (PRR) FLAGELLIN-SENSITIVE 2 (FLS2), creating thus a physical link between PTI and ETI (Qi et al., 2011). The *rin4* null mutation lethality is rescued in a *rin4rps2* double mutant, indicating that RIN4 negatively regulates inappropriate activation of RPS2 (Mackey et al., 2003). In addition, fragments of RIN4, including those produced by AvrRpt2, each containing a nitrate-induced (NOI) domain specific for plants, suppress PTI, also in the *rpm1*/*rps2*/*rin4* mutant background, and activate a cell death response in the wild type (Afzal et al., 2011).

## Membrane Trafficking and the R Proteins-Dependent Immunity

Surprisingly, several basic regulators expected to function in the endomembrane trafficking and membrane fusion events, such as SNARE and exocyst proteins, might be also connected to the regulation of activity of R proteins. For instance, the dwarf and lesion-mimic double mutant of plasma membrane syntaxins SYP121 and SYP122 constitutively expresses the SA signaling pathway- as well as other known pathogen-responsive genes (Zhang et al., 2008). The same study shows that based on the suppressor mutant analysis of *syp121 syp122*, PAD4 is of key importance for the lesion development. Mutant alleles of signaling mediators of both TNL and CNL-type resistances *EDS1, NDR1, REQUIRED FOR MLO12 RESISTANCE 1* (*RAR1*) and *SUPPRESSOR OF G-TWO ALLELE OF SKP1* (*SGT1b*) partially rescued the lesion-mimic phenotype. Interestingly, the double mutant was crossed to the autophagy *atg7* mutant, however, as there was no effect of this mutation on the appearance of lesions, authors concluded that the autophagy does not play a role in this process (Azevedo et al., 2002; Zhang et al., 2008).

Recently, *exo70B1* loss-of-function mutant was found to develop spontaneous leaf lesions, over-express defense responses genes and show enhanced resistance to fungal, oomycete and bacterial pathogens (Kulich et al., 2013; Stegmann et al., 2013). Unexpectedly, its function is not related to the secretion of secretory vesicles to the plasma membrane; instead, EXO70B1 positive compartments were found to end in the central vacuole and to co-localize with autophagosomal marker ATG8f. In a screen for mutants that suppress *exo70B1* phenotype, nine alleles of TIR-NBS2 (TN2) were identified, suggesting that loss-of-function of EXO70B1 leads to activation of this TN protein (Zhao et al., 2015). It was also shown that TN2 interacts with EXO70B1 in yeast and *in planta*. However, it is not known whether TN2 directly monitors EXO70B1 integrity (as proposed by Zhao et al., 2015) or whether EXO70B1 is only required for autophagic transport to the vacuole and subsequent degradation of TN2. EXO70B1-mediated autophagy-related transport to the vacuole might be participating in TN2 degradation. Both scenarios would explain the observed phenotype.

Additionally, recent work confirmed the importance of membrane trafficking in the plant cell death lesion suppression – *lazarus 4* (*laz4*) was found to be mutated in one of three *VACUOLAR PROTEIN SORTING 35* (*VPS35*) genes which code for a subunit of the retromer complex functioning in endosomal protein sorting and vacuolar trafficking – esp. of retrograde retrieval of vacuolar sorting receptors. These results also showed that the retromer deficiency impairs endosomal sorting of immune components and targeting of vacuolar cargo (Munch et al., 2015).

Interestingly, the endosomal compartment may be as well the site of R-Avr proteins interaction – potato R3A and *Phytophtora infestans* effector AVR3a interact and relocalize from the cytoplasm to endocytotic compartment from where they turn on HR signaling (Engelhardt et al., 2012).

Even though it was not described for plants so far, we can expect that the both endosomes and autophagy related membrane trafficking will provide pathogens an opportunity to manipulate both for the purposes of more successful infection. Such an example was recently described for human epithelium-*Salmonella* interaction – at early stages of *S. typhimurium* infection, autophagy is used to seal endosomal membranes damaged by *Salmonella* secretion system during host cell invasion, but later it is also necessary for the further progression of *Salmonella* infection (Kreibich et al., 2015).

## From Autophagy to Immunity

Autophagy is a bulk degradation by which cell/organism recycles nutrients, deals with stress, clears off dysfunctional organelles, aggregates etc. (Levine and Klionsky, 2004). Several types of autophagy have been reported, including macroautophagy, which is present in many organisms including fungi, animals and plants. This process relies on the concerted action of autophagy-related (*ATG*) genes encoded proteins to form first phagophore, to promote phagophore enclosure into autophagosome, and to deliver autophagosomes to the vacuole or lysosomes to release the autophagic bodies for eventual breakdown (Li and Vierstra, 2012; Reggiori and Klionsky, 2013).

When *Arabidopsis* mutants are disrupted in *ATG* genes represented by single loci, they grow normally under non-stress conditions, but are hypersensitive to nitrogen and carbon starvation (Doelling et al., 2002; Hanaoka et al., 2002; Yoshimoto et al., 2004; Thompson et al., 2005). However, unlike other non-plant organisms, *Arabidopsis* has nine *ATG8* and two *ATG12* gene isoforms, which makes the study of their role more difficult and suggests that the autophagic process in plants is more complicated than in other organisms. Some of its complexity is reflected in the role of autophagy in plant immunity.

The importance of autophagy in the plant immunity was first demonstrated in Liu et al. (2005) – it was found that the autophagy was required to restrict the spread of plant HR cell death. The activation of hypersensitive cell death via the R gene RPM1 upon infection with bacteria also led to cell death beyond the borders of the infection site in plants silenced for *atg6/Beclin1* (Patel and Dinesh-Kumar, 2008). It was concluded that autophagy prevents unrestricted HR cell death and that functions as a pro-survival pathway in plant–pathogen interactions. All of these observations and conclusions were based on experimenting with older *Arabidopsis* plants and on tissues surrounding the actual infection sites, a few days after local infection.

However, a pro-death function of autophagy during HR cell death was reported as well (Hofius et al., 2009). Autophagy was found to be triggered by some, but not all types of R proteins in the infected tissue and its surroundings. HR cell death triggered by R proteins RPS4, RPP1 and RPM1 was significantly suppressed in *atg* (autophagy) mutants; especially the first two of them which signal through EDS1 signaling component. In this case, cell death was monitored in the actual infection site, in the range of hours after inoculation (Hofius et al., 2009).

Yoshimoto et al. (2009), found no deviations in RPM1-triggered cell death beyond the initial infection site in younger atg mutants. However, in older *atg* mutants such as *atg5*, they observed lesions in non-infected tissues 6–9 days after infection. Interestingly, these effects were suppressed by removal of the SA and by mutations in SA signaling hub “non-expressor of *PR* genes” – NPR1. The authors proposed that autophagy negatively regulates the cell death by controlling NPR1-dependent SA signaling. In contrast to younger leaves, older *atg* mutant leaves contain higher levels of toxic metabolites, disrupted organelles and oxidized proteins which contribute to the cell death spread (Yoshimoto et al., 2009). This could be as well explained as a combination of effects of different sets of genes involved in the adult plant resistance and ineffective autophagy (Carviel et al., 2009). Scientists tried to explain and integrate these conflicting results obtained from studies on HR lesions of *atg* mutants. Zhou et al. (2014) propose that autophagy suppresses SA and ROS signaling amplification loop that leads to cell death, while in the resistance to necrotrophic pathogens it promotes JA signaling. Consistently, a recent hypothesis suggests that SA is not only an autophagy inducer, but also a cargo for autophagy-related ER to vacuole membrane transport and catabolism (Kulich and Zarsky, 2014). Recently, a model was worked out in which the autophagy is both initiator and executioner of cell death and is placed downstream of the R protein activation, and supposed to help the cell to deal with the ER stress provoked by a heavy load with pathogenesis related proteins (PRs, review in Minina et al., 2014).

## Conclusion and Perspectives

**FIGURE 1:**
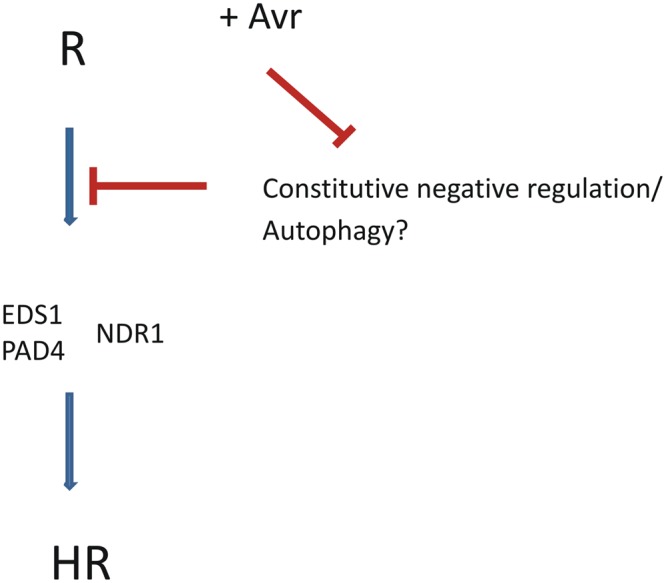
**The plant constitutively negatively regulates R protein function.** The interaction R-Avr serves mainly to release this negative regulation. The important component of negative regulation is autophagy; when it is non-efficient, R proteins spontaneously over activate defense responses.

Here we show that most of the observed defects in *Arabidopsis* R protein regulator mutants are a direct or indirect consequence of non-pathogen related ectopic R protein activation. It thus seems conceivable that the plant constantly down regulates R protein function, and when this constitutive negative regulation is disturbed, the R proteins are activated and spontaneously signal the non-existent pathogen attack. Based on the example of *rin4* mutant lethality we could speculate, that, similarly to other organisms, the proper function of the negative control might be set already in the earliest stages of development. The plant innate immunity has to be kept as low as possible when it is not necessary in order to prevent high energy costs of defense, and yet in the state of alertness which will allow its fast, in fact instantaneous, activation. We believe that the best way to achieve this is to keep these components (i.e., in our case R genes) transcribed and translated on a sufficient basic level, but to keep their function tightly under negative control which will prevent undesired overactive autoimmunity. How could be this achieved? There are many examples of negative controls involved at various stages of defense that include ubiquitination and proteolysis, phosphorylation of proteins, as well as redox dependent changes in protein multimerization and localization (e.g., Trujillo et al., 2008; Anderson et al., 2011; Vogelmann et al., 2012). We suggest that one of the mechanisms to achieve this is also targeting of defense machinery components – here especially R proteins – to the autophagy pathway for degradation (**Figure 1**). Once the R protein is recruited by autophagy machinery into the autophagosome, it might share the destiny of other autophagic cargos – transport to the vacuole and degradation. We speculate that along with proteins the autophagy related degradation process might destroy also other molecules including signaling relevant molecules as ROS or SA. After the interaction of R protein with its counterpart Avr, R protein is protected against this autophagy-dependent degradation and can interact with downstream components and trigger ETI. This model may be valid also for indirect R-Avr interactions; e.g., the proposed R protein guard function (reviewed e. g. in Spoel and Dong, 2012) could be based on the avoidance of this negative regulation after the recognition of the changed status of the guardee. It should be stressed that we certainly expect other ways of regulations of R proteins to exist, such as a switch from inactive to active state of R protein upon Avr recognition, as well as other ways of negative regulation.

Based on our hypothesis one would expect that autophagy mutants should copy the phenotype of *exo70B1* mutant, having at least some R proteins constitutively activated. While some of the mutants in the autophagy pathway indeed show similar phenotypic deviations (e.g., early senescence and yellowing, sensitivity to starvation, as well as SA hyperaccumulation in *atg2* and *atg5* mutants; Yoshimoto et al., 2009; Wang et al., 2011), others seem to display only early senescence phenotype and cell death phenotypes only after starvation induction (like *atg7* mutant). It also seems that some subunits of autophagy machinery might be more important for the negative regulation of the immunity, while others, e.g., ATG7 and ATG9, in the execution of HR (Hofius et al., 2009; Minina et al., 2014). It should be, however, noted that autophagy proteins (and EXO70B1) have been also implicated in diverse cellular processes independently of their roles in autophagy.

We also expect that, pathogen effectors might have evolved to manipulate and hijack this negative regulation and worsen the plant defense – recently, a *Phytophtora infestans* effector PexRD54 has been shown to outcompete the autophagy cargo receptor Joka and enhance virulence of this pathogen. Interestingly, PexRD54 does this probably through the activation of selective autophagy. Joka could participate in the removal of plant or pathogen molecules that negatively affect host defenses. As authors of the study speculate, PexRD54 would thus counteract the positive role of Joka2-mediated selective autophagy in pathogen defense. An alternative, but not exclusive explanation based on our hypothesis would be that PexRD54 at the same time stimulates the selective autophagy of R proteins capable of detecting it and thus promotes pathogen virulence (Dagdas et al., 2016). Already the report of Engelhardt et al. (2012) demonstrated the capability of cytoplasmic R protein to be recruited to endomembranes, but not for degradation, rather for the purpose of activation. However, this is not exclusive with our model – the interaction of R3A and Avr3A might release the negative regulation of R3A and switch on the HR. This interaction is obviously indirect and requires an intermediate connected to ARA6/ARA7 marked endosomes. It is possible that this activation evolved from the mechanisms of negative regulation. More information on R3A and Avr3A interactors could help to solve this ambivalent situation.

We found an indirect support for our hypothesis in the autopagy-related events described for mammalian cells – it is known from experiments performed on HeLa cells that endocytosed plasma membrane contributes to ATG12–ATG5-ATG16L1-positive/ATG8-negative phagophore precursor vesicles by both clathrin-dependent and -independent routes (Moreau and Rubinsztein, 2012). The subsequent maturation of these small phagophore precursors into phagophores (ATG12–ATG5-ATG16L1-positive/ATG8-positive) is assisted by SNARE-mediated homotypic fusion that increases their size. Additionally, *Arabidopsis* BON1/2/3 belong to copine proteins, a family of ubiquitous Ca(2+)-dependent, phospholipid-binding proteins that are known to be involved in animal membrane trafficking events (Tomsig and Creutz, 2002), and in *Dictyostelium* localize to plasma membrane, contractile vacuoles, organelles of the endolysosomal pathway, and phagosomes (Damer et al., 2005). Therefore, besides confirmed role of EXO70B1 in autophagy and regulation of TN2 activity, very probably SNARE and BON proteins could implement similar role in autophagy-related membrane targeting and membrane fusion events leading to the negative control of R proteins.

**FIGURE 2:**
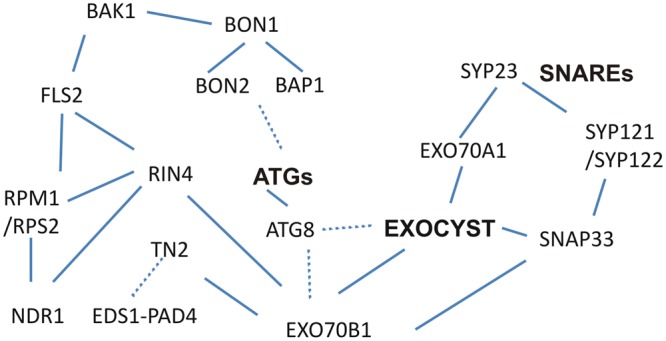
**Interactions of PTI, ETI and ATG related proteins with SNAREs and exocyst.** The RIN4 interacts with PTI receptor FLS2 and associated proteins, which further interact with BON1, BON2 and BAP. Additionally, RIN4 and related R-proteins may be in the same complex. R proteins and autophagy functions are connected by exocyst and SNARE interactions. (Experimentally confirmed interactions are visualized with lines, non-confirmed with dashed lines).

We found further support for this hypothesis in the connection between assumed autophagy regulating proteins and R proteins, as well as other key molecules of the both PTI and ETI immune response, in the web of protein-protein interactions that are available in Biogrid and PPIN databases (Stark et al., 2006; Mukhtar et al., 2011; **Figure 2**; **Table 1**). The components of PAMP-sensing complexes interact with RIN4, which further interacts with R proteins. Mainly through mediating kinase BAK1, they are connected and interact as well with BON1, BON2 and BAP. RIN4 interacts with R proteins as well as with EXO70B1 (Afzal et al., 2013). Besides its capability to interact with other exocyst and SNARE proteins, EXO70B1, together with 20 other paralogs of *Arabidopsis* EXO70 exocyst subunits, possess ATG8 interacting motives, which indicates that the autophagy machinery and exocyst complex functions are multiply connected (Cvrčková and Zárský, 2013; Tzfadia and Galili, 2013; Sabol et al., in preparation). Thus, in the vicinity of plasma membrane, and depending on membrane trafficking which involves SNARE, exocyst and autophagy complex proteins, a tight control of R protein activation allows the immunity to be kept low but in a constant alert.

Recently, a role for EXO70F3 of *Oryza sativa* in immunity against *Magnaporthe oryzae* was found – OsEXO70F3 appears to play a crucial role in immunity triggered by Pii, suggesting a role for this EXO70 paralog as a decoy or helper in Pii/Avr-Pii interaction (Fujisaki et al., 2015). It may be true that pathogen effectors target these and other exocyst subunits in order to suppress defense, however, we don’t consider it to be mutually exclusive with our hypothesis.

Our model could help to better understand and reconcile conflicting aspects of autophagy in the plant immunity (Teh and Hofius, 2014): in the infection sites, R-Avr recognition prevents R protein targeting to inactivation/destruction pathway and triggers the ETI, and with the increased distance from the infection site, declining concentration of Avr protein allows the autophagy to overtake again a control over R protein. In *atg* mutants, the existing constitutive immunity activation results in spontaneous HR lesions formation; but after the pathogen attack, in the case of younger leaves, in addition to R protein deregulation, R is further activated by Avr recognition, which makes cells more resistant and lesions smaller. Or, under conditions with additional stresses, as in the case also of older leaves, because of coincidence between consequences of ineffective autophagy of *atg* mutants and Avr-enhanced over activation of R proteins, less Avr is needed for HR threshold to be crossed and lesions spread farther.

Our model’s aim is to focus on one aspect only – a possibility of a negative regulation of some NLRs/innate immunity related proteins by autophagy in plants. However, there are many difficulties that will have to be overcome in order to confirm its validity. Part of difficulties is coming from the complexity of autophagy machinery and a large number of ATG proteins that have also been implicated in diverse cellular processes independently of their roles in autophagy. Autophagy machinery is also difficult to study separately from other endomembrane compartments, especially by using pharmacological treatments. For instance, wortmannin, which is often used for these purposes, is rather pleiotropic drug – dependent on cell type and concentration it affects different types of phosphoinositide kinases, having thus multiple interference with endomembrane dynamics.

To conclude, plants have mechanisms to downregulate R proteins function, and when they are attacked by an appropriate Avr carrying pathogen, the R proteins are stabilized, activating defense responses. This would also mean that R proteins are capable of immunity activation without Avr and that the interaction R-Avr serves mainly to release R proteins negative regulation. The disturbance of the basic autophagy machinery has pleiotropic effects on many plant functions including development and is influenced by growth conditions, abiotic stresses and senescence, hence it is very difficult to study effects of *atg* mutants that would concern specifically defense responses. We believe that in the near future, R proteins studies will bring more information on the regulation of their activity including proteins that target them for the suggested autophagy destruction.

## Author Contributions

TP did a compilation of data on R-related dwarf and lesion mimic mutants and most of the writing; PS did the RIN4-related data mining and text editing; IK explained the connection to autophagy; JO delt with membrane traficking chapter; VZ did the most of text editing and integrating as well as the finalization of the manuscript.

## Conflict of Interest Statement

The authors declare that the research was conducted in the absence of any commercial or financial relationships that could be construed as a potential conflict of interest.

The reviewer TOB and handling Editor declared their shared affiliation, and the handling Editor states that the process nevertheless met the standards of a fair and objective review

## References

[B1] Afzal A. J. da Cunha L. Mackey D. (2011). Separable fragments and membrane tethering of *Arabidopsis* RIN4 regulate its suppression of PAMP-triggered immunity. *Plant Cell* 23 3798–3811. 10.1105/tpc.111.088708 21984695 PMC3229150

[B2] Afzal A. J. Kim J. H. Mackey D. (2013). The role of NOI-domain containing proteins in plant immune signaling. *BMC Genomics* 14:327 10.1186/1471-2164-14-327 PMC3661340 23672422

[B3] Anderson J. C. Bartels S. Gonzalez Besteiro M. A. Shahollari B. Ulm R. Peck S. C. (2011). *Arabidopsis* MAP Kinase Phosphatase 1 (AtMKP1) negatively regulates MPK6-mediated PAMP responses and resistance against bacteria. *Plant J.* 67 258–268. 10.1111/j.1365-313X.2011.04588.x 21447069

[B4] Axtell M. J. Staskawicz B. J. (2003). Initiation of RPS2-specified disease resistance in *Arabidopsis* is coupled to the AvrRpt2-directed elimination of RIN4. *Cell* 112 369–377. 10.1016/S0092-8674(03)00036-9 12581526

[B5] Azevedo C. Sadanandom A. Kitagawa K. Freialdenhoven A. Shirasu K. Schulze-Lefert P. (2002). The RAR1 interactor SGT1, an essential component of R gene-triggered disease resistance. *Science* 295 2073–2076. 10.1126/science.1067554 11847307

[B6] Bartels S. Anderson J. C. Gonzalez Besteiro M. A. Carreri A. Hirt H. Buchala A. (2009). MAP kinase phosphatase1 and protein tyrosine phosphatase1 are repressors of salicylic acid synthesis and SNC1-mediated responses in *Arabidopsis*. *Plant Cell* 21 2884–2897. 10.1105/tpc.109.067678 19789277 PMC2768924

[B7] Bonardi V. Tang S. Stallmann A. Roberts M. Cherkis K. Dangl J. L. (2011). Expanded functions for a family of plant intracellular immune receptors beyond specific recognition of pathogen effectors. *Proc. Natl. Acad. Sci. U.S.A.* 108 16463–16468. 10.1073/pnas.1113726108 21911370 PMC3182704

[B8] Bowling S. A. Guo A. Cao H. Gordon A. S. Klessig D. F. Dong X. (1994). A mutation in *Arabidopsis* that leads to constitutive expression of systemic acquired resistance. *Plant Cell* 6 1845–1857. 10.1105/tpc.6.12.1845 7866028 PMC160566

[B9] Brodersen P. Petersen M. Pike H. M. Olszak B. Skov S. Odum N. (2002). Knockout of *Arabidopsis* accelerated-cell-death11 encoding a sphingosine transfer protein causes activation of programmed cell death and defense. *Genes Dev.* 16 490–502. 10.1101/gad.218202 11850411 PMC155338

[B10] Carviel J. L. Al-Daoud F. Neumann M. Mohammad A. Provart N. J. Moeder W. (2009). Forward and reverse genetics to identify genes involved in the age-related resistance response in *Arabidopsis thaliana*. *Mol. Plant Pathol.* 10 621–634. 10.1111/j.1364-3703.2009.00557.x 19694953 PMC6640485

[B11] Century K. S. Holub E. B. Staskawicz B. J. (1995). NDR1, a locus of *Arabidopsis thaliana* that is required for disease resistance to both a bacterial and a fungal pathogen. *Proc. Natl. Acad. Sci. U.S.A.* 92 6597–6601. 10.1073/pnas.92.14.6597 11607554 PMC41565

[B12] Century K. S. Shapiro A. D. Repetti P. P. Dahlbeck D. Holub E. Staskawicz B. J. (1997). NDR1, a pathogen-induced component required for *Arabidopsis* disease resistance. *Science* 278 1963–1965. 10.1126/science.278.5345.1963 9395402

[B13] Chatre L. Wattelet-Boyer V. Melser S. Maneta-Peyret L. Brandizzi F. Moreau P. (2009). A novel di-acidic motif facilitates ER export of the syntaxin SYP31. *J. Exp. Bot.* 60 3157–3165. 10.1093/jxb/erp155 19516076 PMC2718219

[B14] Collier S. M. Hamel L. P. Moffett P. (2011). Cell death mediated by the N-terminal domains of a unique and highly conserved class of NB-LRR protein. *Mol. Plant Microbe Interact.* 24 918–931. 10.1094/MPMI-03-11-0050 21501087

[B15] Cvrčková F. Zárský V. (2013). Old AIMs of the exocyst: evidence for an ancestral association of exocyst subunits with autophagy-associated Atg8 proteins. *Plant Signal. Behav.* 8 e27099. 10.4161/psb.27099 PMC4091244 24305598

[B16] Dagdas Y. F. Belhaj K. Maqbool A. Chaparro-Garcia A. Pandey P. Petre B. (2016). An effector of the Irish potato famine pathogen antagonizes a host autophagy cargo receptor. *Elife* 5 e10856 10.7554/eLife.10856 PMC4775223 26765567

[B17] Damer C. K. Bayeva M. Hahn E. S. Rivera J. Socec C. I. (2005). Copine A, a calcium-dependent membrane-binding protein, transiently localizes to the plasma membrane and intracellular vacuoles in *Dictyostelium*. *BMC Cell Biol.* 6:46 10.1186/1471-2121-6-46 PMC1327671 16343335

[B18] Debener T. Lehnackers H. Arnold M. Dangl J. L. (1991). Identification and molecular mapping of a single *Arabidopsis thaliana* locus determining resistance to a phytopathogenic *Pseudomonas syringae* isolate. *Plant J.* 1 289–302. 10.1046/j.1365-313X.1991.t01-7-00999.x 21736648

[B19] Doelling J. H. Walker J. M. Friedman E. M. Thompson A. R. Vierstra R. D. (2002). The APG8/12-activating enzyme APG7 is required for proper nutrient recycling and senescence in *Arabidopsis thaliana*. *J. Biol. Chem.* 277 33105–33114. 10.1074/jbc.M204630200 12070171

[B20] Engelhardt S. Boevink P. C. Armstrong M. R. Ramos M. B. Hein I. Birch P. R. (2012). Relocalization of late blight resistance protein R3a to endosomal compartments is associated with effector recognition and required for the immune response. *Plant Cell* 24 5142–5158. 10.1105/tpc.112.104992 23243124 PMC3556980

[B21] Feys B. J. Moisan L. J. Newman M. A. Parker J. E. (2001). Direct interaction between the *Arabidopsis* disease resistance signaling proteins, EDS1 and PAD4. *EMBO J.* 20 5400–5411. 10.1093/emboj/20.19.5400 11574472 PMC125652

[B22] Feys B. J. Wiermer M. Bhat R. A. Moisan L. J. Medina-Escobar N. Neu C. (2005). *Arabidopsis* SENESCENCE-ASSOCIATED GENE101 stabilizes and signals within an ENHANCED DISEASE SUSCEPTIBILITY1 complex in plant innate immunity. *Plant Cell* 17 2601–2613. 10.1105/tpc.105.033910 16040633 PMC1197438

[B23] Fujiki Y. Yoshimoto K. Ohsumi Y. (2007). An *Arabidopsis* homolog of yeast ATG6/VPS30 is essential for pollen germination. *Plant Physiol.* 143 1132–1139. 10.1104/pp.106.093864 17259285 PMC1820928

[B24] Fujisaki K. Abe Y. Ito A. Saitoh H. Yoshida K. Kanzaki H. (2015). Rice Exo70 interacts with a fungal effector, AVR-Pii, and is required for AVR-Pii-triggered immunity. *Plant J.* 83 875–887. 10.1111/tpj.12934 26186703

[B25] Gao M. Wang X. Wang D. Xu F. Ding X. Zhang Z. (2009). Regulation of cell death and innate immunity by two receptor-like kinases in *Arabidopsis*. *Cell Host Microbe* 6 34–44. 10.1016/j.chom.2009.05.019 19616764

[B26] Gomez-Gomez L. Boller T. (2000). FLS2: an LRR receptor-like kinase involved in the perception of the bacterial elicitor flagellin in *Arabidopsis*. *Mol. Cell* 5 1003–1011. 10.1016/S1097-2765(00)80265-8 10911994

[B27] Gou M. Hua J. (2012). Complex regulation of an R gene SNC1 revealed by auto-immune mutants. *Plant Signal. Behav.* 7 213–216. 10.4161/psb.18884 22415045 PMC3405709

[B28] Gou M. Shi Z. Zhu Y. Bao Z. Wang G. Hua J. (2012). The F-box protein CPR1/CPR30 negatively regulates R protein SNC1 accumulation. *Plant J.* 69 411–420. 10.1111/j.1365-313X.2011.04799.x 21967323

[B29] Grant J. J. Chini A. Basu D. Loake G. J. (2003). Targeted activation tagging of the *Arabidopsis* NBS-LRR gene, ADR1, conveys resistance to virulent pathogens. *Mol. Plant Microbe Interact.* 16 669–680. 10.1094/MPMI.2003.16.8.669 12906111

[B30] Hanaoka H. Noda T. Shirano Y. Kato T. Hayashi H. Shibata D. (2002). Leaf senescence and starvation-induced chlorosis are accelerated by the disruption of an *Arabidopsis* autophagy gene. *Plant Physiol.* 129 1181–1193. 10.1104/pp.011024 12114572 PMC166512

[B31] He Y. Gan S. (2002). A gene encoding an acyl hydrolase is involved in leaf senescence in *Arabidopsis*. *Plant Cell* 14 805–815. 10.1105/tpc.010422 11971136 PMC150683

[B32] Hofius D. Schultz-Larsen T. Joensen J. Tsitsigiannis D. I. Petersen N. H. Mattsson O. (2009). Autophagic components contribute to hypersensitive cell death in *Arabidopsis*. *Cell* 137 773–783. 10.1016/j.cell.2009.02.036 19450522

[B33] Hua J. Grisafi P. Cheng S. H. Fink G. R. (2001). Plant growth homeostasis is controlled by the *Arabidopsis* BON1 and BAP1 genes. *Genes Dev.* 15 2263–2272. 10.1101/gad.918101 11544183 PMC312777

[B34] Huang X. Li J. Bao F. Zhang X. Yang S. (2010). A gain-of-function mutation in the *Arabidopsis* disease resistance gene RPP4 confers sensitivity to low temperature. *Plant Physiol.* 154 796–809. 10.1104/pp.110.157610 20699401 PMC2949010

[B35] Jacob F. Vernaldi S. Maekawa T. (2013). Evolution and conservation of plant NLR functions. *Front. Immunol.* 4:297 10.3389/fimmu.2013.00297 PMC3782705 24093022

[B36] Janda M. Ruelland E. (2014). Magical mystery tour: salicylic acid signaling. *Environ. Exp. Bot.* 114 117–128. 10.1016/j.envexpbot.2014.07.003

[B37] Jirage D. Tootle T. L. Reuber T. L. Frost L. N. Feys B. J. Parker J. E. (1999). *Arabidopsis thaliana* PAD4 encodes a lipase-like gene that is important for salicylic acid signaling. *Proc. Natl. Acad. Sci. U.S.A.* 96 13583–13588. 10.1073/pnas.96.23.13583 10557364 PMC23991

[B38] Jones J. D. Dangl J. L. (2006). The plant immune system. *Nature* 444 323–329. 10.1038/nature05286 17108957

[B39] Kato H. Saito T. Ito H. Komeda Y. Kato A. (2014). Overexpression of the TIR-X gene results in a dwarf phenotype and activation of defense-related gene expression in *Arabidopsis thaliana*. *J. Plant Physiol.* 171 382–388. 10.1016/j.jplph.2013.12.002 24594389

[B40] Ketelaar T. Voss C. Dimmock S. A. Thumm M. Hussey P. J. (2004). *Arabidopsis* homologues of the autophagy protein Atg8 are a novel family of microtubule binding proteins. *FEBS Lett.* 567 302–306. 10.1016/j.febslet.2004.04.088 15178341

[B41] Kim H. S. Desveaux D. Singer A. U. Patel P. Sondek J. Dangl J. L. (2005). The *Pseudomonas syringae* effector AvrRpt2 cleaves its C-terminally acylated target, RIN4, from *Arabidopsis* membranes to block RPM1 activation. *Proc. Natl. Acad. Sci. U.S.A.* 102 6496–6501. 10.1073/pnas.0500792102 15845764 PMC1088372

[B42] Kim S. H. Gao F. Bhattacharjee S. Adiasor J. A. Nam J. C. Gassmann W. (2010). The *Arabidopsis* resistance-like gene SNC1 is activated by mutations in SRFR1 and contributes to resistance to the bacterial effector AvrRps4. *PLoS Pathog.* 6:e1001172 10.1371/journal.ppat.1001172 PMC2973837 21079790

[B43] Kliebenstein D. J. Dietrich R. A. Martin A. C. Last R. L. Dangl J. L. (1999). LSD1 regulates salicylic acid induction of copper zinc superoxide dismutase in *Arabidopsis thaliana*. *Mol. Plant Microbe Interact.* 12 1022–1026. 10.1094/MPMI.1999.12.11.1022 10550898

[B44] Kong Q. Qu N. Gao M. Zhang Z. Ding X. Yang F. (2012). The MEKK1-MKK1/MKK2-MPK4 kinase cascade negatively regulates immunity mediated by a mitogen-activated protein kinase kinase kinase in *Arabidopsis*. *Plant Cell* 24 2225–2236. 10.1105/tpc.112.097253 22643122 PMC3442598

[B45] Kreibich S. Emmenlauer M. Fredlund J. Ramo P. Munz C. Dehio C. (2015). Autophagy proteins promote repair of endosomal membranes damaged by the *Salmonella* type three secretion system 1. *Cell Host Microbe* 18 527–537. 10.1016/j.chom.2015.10.015 26567507

[B46] Kulich I. Pecenkova T. Sekeres J. Smetana O. Fendrych M. Foissner I. (2013). *Arabidopsis* exocyst subcomplex containing subunit EXO70B1 is involved in autophagy-related transport to the vacuole. *Traffic* 14 1155–1165. 10.1111/tra.12101 23944713

[B47] Kulich I. Zarsky V. (2014). Autophagy-related direct membrane import from ER/cytoplasm into the vacuole or apoplast: a hidden gateway also for secondary metabolites and phytohormones? *Int. J. Mol. Sci.* 15 7462–7474. 10.3390/ijms15057462 24786101 PMC4057683

[B48] Levine B. Klionsky D. J. (2004). Development by self-digestion: molecular mechanisms and biological functions of autophagy. *Dev. Cell* 6 463–477. 10.1016/S1534-5807(04)00099-1 15068787

[B49] Li F. Vierstra R. D. (2012). Regulator and substrate: dual roles for the ATG1-ATG13 kinase complex during autophagic recycling in *Arabidopsis*. *Autophagy* 8 982–984. 10.4161/auto.20240 22714291 PMC3427266

[B50] Li J. Wen J. Lease K. A. Doke J. T. Tax F. E. Walker J. C. (2002). BAK1, an *Arabidopsis* LRR receptor-like protein kinase, interacts with BRI1 and modulates brassinosteroid signaling. *Cell* 110 213–222. 10.1016/S0092-8674(02)00812-7 12150929

[B51] Li Y. Pennington B. O. Hua J. (2009). Multiple R-like genes are negatively regulated by BON1 and BON3 in *Arabidopsis*. *Mol. Plant Microbe Interact.* 22 840–848. 10.1094/MPMI-22-7-0840 19522566

[B52] Li Y. Tessaro M. J. Li X. Zhang Y. (2010). Regulation of the expression of plant resistance gene SNC1 by a protein with a conserved BAT2 domain. *Plant Physiol.* 153 1425–1434. 10.1104/pp.110.156240 20439546 PMC2899940

[B53] Liu J. Liu X. Dai L. Wang G. (2007). Recent progress in elucidating the structure, function and evolution of disease resistance genes in plants. *J. Genet. Genomics* 34 765–776. 10.1016/S1673-8527(07)60087-3 17884686

[B54] Liu Y. Schiff M. Czymmek K. Talloczy Z. Levine B. Dinesh-Kumar S. P. (2005). Autophagy regulates programmed cell death during the plant innate immune response. *Cell* 121 567–577. 10.1016/j.cell.2005.03.007 15907470

[B55] Lorrain S. Vailleau F. Balague C. Roby D. (2003). Lesion mimic mutants: keys for deciphering cell death and defense pathways in plants? *Trends Plant Sci.* 8 263–271. 10.1016/S1360-1385(03)00108-0 12818660

[B56] Mackey D. Belkhadir Y. Alonso J. M. Ecker J. R. Dangl J. L. (2003). *Arabidopsis* RIN4 is a target of the type III virulence effector AvrRpt2 and modulates RPS2-mediated resistance. *Cell* 112 379–389. 10.1016/S0092-8674(03)00040-0 12581527

[B57] Mackey D. Holt B. F. III Wiig A. Dangl J. L. (2002). RIN4 interacts with *Pseudomonas syringae* type III effector molecules and is required for RPM1-mediated resistance in *Arabidopsis*. *Cell* 108 743–754. 10.1016/S0092-8674(02)00661-X 11955429

[B58] Martin G. B. (1999). Functional analysis of plant disease resistance genes and their downstream effectors. *Curr. Opin. Plant Biol.* 2 273–279. 10.1016/S1369-5266(99)80049-1 10458999

[B59] McDowell J. M. Woffenden B. J. (2003). Plant disease resistance genes: recent insights and potential applications. *Trends Biotechnol.* 21 178–183. 10.1016/S0167-7799(03)00053-2 12679066

[B60] Meyers B. C. Kozik A. Griego A. Kuang H. Michelmore R. W. (2003). Genome-wide analysis of NBS-LRR-encoding genes in *Arabidopsis*. *Plant Cell* 15 809–834. 10.1105/tpc.009308 12671079 PMC152331

[B61] Minina E. A. Bozhkov P. V. Hofius D. (2014). Autophagy as initiator or executioner of cell death. *Trends Plant Sci.* 19 692–697. 10.1016/j.tplants.2014.07.007 25156061

[B62] Moreau K. Rubinsztein D. C. (2012). The plasma membrane as a control center for autophagy. *Autophagy* 8 861–863. 10.4161/auto.20060 22617437 PMC3378426

[B63] Mukhtar M. S. Carvunis A. R. Dreze M. Epple P. Steinbrenner J. Moore J. (2011). Independently evolved virulence effectors converge onto hubs in a plant immune system network. *Science* 333 596–601. 10.1126/science.1203659 21798943 PMC3170753

[B64] Munch D. Teh O. K. Malinovsky F. G. Liu Q. Vetukuri R. R. El Kasmi F. (2015). Retromer contributes to immunity-associated cell death in *Arabidopsis*. *Plant Cell* 27 463–479. 10.1105/tpc.114.132043 25681156 PMC4456924

[B65] Nandety R. S. Caplan J. L. Cavanaugh K. Perroud B. Wroblewski T. Michelmore R. W. (2013). The role of TIR-NBS and TIR-X proteins in plant basal defense responses. *Plant Physiol.* 162 1459–1472. 10.1104/pp.113.219162 23735504 PMC3707564

[B66] Nawrath C. Metraux J. P. (1999). Salicylic acid induction-deficient mutants of *Arabidopsis* express PR-2 and PR-5 and accumulate high levels of camalexin after pathogen inoculation. *Plant Cell* 11 1393–1404. 10.2307/3870970 10449575 PMC144293

[B67] Noutoshi Y. Ito T. Seki M. Nakashita H. Yoshida S. Marco Y. (2005). A single amino acid insertion in the WRKY domain of the *Arabidopsis* TIR-NBS-LRR-WRKY-type disease resistance protein SLH1 (sensitive to low humidity 1) causes activation of defense responses and hypersensitive cell death. *Plant J.* 43 873–888. 10.1111/j.1365-313X.2005.02500.x 16146526

[B68] Ohtomo I. Ueda H. Shimada T. Nishiyama C. Komoto Y. Hara-Nishimura I. (2005). Identification of an allele of VAM3/SYP22 that confers a semi-dwarf phenotype in *Arabidopsis thaliana*. *Plant Cell Physiol.* 46 1358–1365. 10.1093/pcp/pci146 15937323

[B69] Palma K. Thorgrimsen S. Malinovsky F. G. Fiil B. K. Nielsen H. B. Brodersen P. (2010). Autoimmunity in *Arabidopsis* acd11 is mediated by epigenetic regulation of an immune receptor. *PLoS Pathog.* 6:e1001137 10.1371/journal.ppat.1001137 PMC2951382 20949080

[B70] Patel S. Dinesh-Kumar S. P. (2008). *Arabidopsis* ATG6 is required to limit the pathogen-associated cell death response. *Autophagy* 4 20–27. 10.4161/auto.5056 17932459

[B71] Petersen M. Brodersen P. Naested H. Andreasson E. Lindhart U. Johansen B. (2000). *Arabidopsis* map kinase 4 negatively regulates systemic acquired resistance. *Cell* 103 1111–1120. 10.1016/S0092-8674(00)00213-0 11163186

[B72] Pontier D. Balague C. Roby D. (1998). The hypersensitive response. A programmed cell death associated with plant resistance. *C. R. Acad. Sci. III* 321 721–734. 10.1016/S0764-4469(98)80013-9 9809204

[B73] Qi Y. Tsuda K. Glazebrook J. Katagiri F. (2011). Physical association of pattern-triggered immunity (PTI) and effector-triggered immunity (ETI) immune receptors in *Arabidopsis*. *Mol. Plant Pathol.* 12 702–708. 10.1111/j.1364-3703.2010.00704.x 21726371 PMC6640369

[B74] Qiu J. L. Zhou L. Yun B. W. Nielsen H. B. Fiil B. K. Petersen K. (2008). *Arabidopsis* mitogen-activated protein kinase kinases MKK1 and MKK2 have overlapping functions in defense signaling mediated by MEKK1, MPK4, and MKS1. *Plant Physiol.* 148 212–222. 10.1104/pp.108.120006 18599650 PMC2528087

[B75] Reggiori F. Klionsky D. J. (2013). Autophagic processes in yeast: mechanism, machinery and regulation. *Genetics* 194 341–361. 10.1534/genetics.112.149013 23733851 PMC3664846

[B76] Roberts M. Tang S. Stallmann A. Dangl J. L. Bonardi V. (2013). Genetic requirements for signaling from an autoactive plant NB-LRR intracellular innate immune receptor. *PLoS Genet.* 9:e1003465 10.1371/journal.pgen.1003465 PMC3636237 23633962

[B77] Rogers E. E. Ausubel F. M. (1997). *Arabidopsis* enhanced disease susceptibility mutants exhibit enhanced susceptibility to several bacterial pathogens and alterations in PR-1 gene expression. *Plant Cell* 9 305–316. 10.1105/tpc.9.3.305 9090877 PMC156920

[B78] Sasek V. Janda M. Delage E. Puyaubert J. Guivarc’h A. Lopez Maseda E. (2014). Constitutive salicylic acid accumulation in pi4kIIIbeta1beta2 *Arabidopsis* plants stunts rosette but not root growth. *New Phytol.* 203 805–816. 10.1111/nph.12822 24758581

[B79] Sekine K. T. Nandi A. Ishihara T. Hase S. Ikegami M. Shah J. (2004). Enhanced resistance to *Cucumber mosaic* virus in the *Arabidopsis thaliana* ssi2 mutant is mediated via an SA-independent mechanism. *Mol. Plant Microbe Interact.* 17 623–632. 10.1094/MPMI.2004.17.6.623 15195945

[B80] Shirano Y. Kachroo P. Shah J. Klessig D. F. (2002). A gain-of-function mutation in an *Arabidopsis* Toll Interleukin1 receptor-nucleotide binding site-leucine-rich repeat type R gene triggers defense responses and results in enhanced disease resistance. *Plant Cell* 14 3149–3162. 10.1105/tpc.005348 12468733 PMC151208

[B81] Spoel S. H. Dong X. (2012). How do plants achieve immunity? Defence without specialized immune cells. *Nat. Rev. Immunol.* 12 89–100. 10.1038/nri3141 22273771

[B82] Stark C. Breitkreutz B. J. Reguly T. Boucher L. Breitkreutz A. Tyers M. (2006). BioGRID: a general repository for interaction datasets. *Nucleic Acids Res.* 34 D535–D539. 10.1093/nar/gkj109 16381927 PMC1347471

[B83] Stegmann M. Anderson R. G. Westphal L. Rosahl S. McDowell J. M. Trujillo M. (2013). The exocyst subunit Exo70B1 is involved in the immune response of *Arabidopsis thaliana* to different pathogens and cell death. *Plant Signal. Behav.* 8 e27421. 10.4161/psb.27421 PMC4091220 24389869

[B84] Synek L. Schlager N. Elias M. Quentin M. Hauser M. T. Zarsky V. (2006). AtEXO70A1, a member of a family of putative exocyst subunits specifically expanded in land plants, is important for polar growth and plant development. *Plant J.* 48 54–72. 10.1111/j.1365-313X.2006.02854.x 16942608 PMC2865999

[B85] Takemoto D. Jones D. A. (2005). Membrane release and destabilization of *Arabidopsis* RIN4 following cleavage by *Pseudomonas syringae* AvrRpt2. *Mol. Plant Microbe Interact.* 18 1258–1268. 10.1094/MPMI-18-1258 16478045

[B86] Teh O. K. Hofius D. (2014). Membrane trafficking and autophagy in pathogen-triggered cell death and immunity. *J. Exp. Bot.* 65 1297–1312. 10.1093/jxb/ert441 24420567

[B87] Thompson A. R. Doelling J. H. Suttangkakul A. Vierstra R. D. (2005). Autophagic nutrient recycling in *Arabidopsis* directed by the ATG8 and ATG12 conjugation pathways. *Plant Physiol.* 138 2097–2110. 10.1104/pp.105.060673 16040659 PMC1183398

[B88] Tomsig J. L. Creutz C. E. (2002). Copines: a ubiquitous family of Ca(2+)-dependent phospholipid-binding proteins. *Cell. Mol. Life Sci.* 59 1467–1477. 10.1007/s00018-002-8522-7 12440769 PMC11337430

[B89] Trujillo M. Ichimura K. Casais C. Shirasu K. (2008). Negative regulation of PAMP-triggered immunity by an E3 ubiquitin ligase triplet in *Arabidopsis*. *Curr. Biol.* 18 1396–1401. 10.1016/j.cub.2008.07.085 18771922

[B90] Tzfadia O. Galili G. (2013). The *Arabidopsis* exocyst subcomplex subunits involved in a golgi-independent transport into the vacuole possess consensus autophagy-associated atg8 interacting motifs. *Plant Signal. Behav.* 8 e26732-1–e26732-3. 10.4161/psb.26732 PMC4091113 24494242

[B91] Vlot A. C. Liu P. P. Cameron R. K. Park S. W. Yang Y. Kumar D. (2008). Identification of likely orthologs of tobacco salicylic acid-binding protein 2 and their role in systemic acquired resistance in *Arabidopsis thaliana*. *Plant J.* 56 445–456. 10.1111/j.1365-313X.2008.03618.x 18643994

[B92] Vogelmann K. Drechsel G. Bergler J. Subert C. Philippar K. Soll J. (2012). Early senescence and cell death in *Arabidopsis* saul1 mutants involves the PAD4-dependent salicylic acid pathway. *Plant Physiol.* 159 1477–1487. 10.1104/pp.112.196220 22706448 PMC3425192

[B93] Wagner S. Stuttmann J. Rietz S. Guerois R. Brunstein E. Bautor J. (2013). Structural basis for signaling by exclusive EDS1 heteromeric complexes with SAG101 or PAD4 in plant innate immunity. *Cell Host Microbe* 14 619–630. 10.1016/j.chom.2013.11.006 24331460

[B94] Wang Y. Nishimura M. T. Zhao T. Tang D. (2011). ATG2, an autophagy-related protein, negatively affects powdery mildew resistance and mildew-induced cell death in *Arabidopsis*. *Plant J.* 68 74–87. 10.1111/j.1365-313X.2011.04669.x 21645148

[B95] Yang H. Shi Y. Liu J. Guo L. Zhang X. Yang S. (2010). A mutant CHS3 protein with TIR-NB-LRR-LIM domains modulates growth, cell death and freezing tolerance in a temperature-dependent manner in *Arabidopsis*. *Plant J.* 63 283–296. 10.1111/j.1365-313X.2010.04241.x 20444230

[B96] Yang S. Hua J. (2004). A haplotype-specific Resistance gene regulated by BONZAI1 mediates temperature-dependent growth control in *Arabidopsis*. *Plant Cell* 16 1060–1071. 10.1105/tpc.020479 15031411 PMC412877

[B97] Yang S. Yang H. Grisafi P. Sanchatjate S. Fink G. R. Sun Q. (2006). The BON/CPN gene family represses cell death and promotes cell growth in *Arabidopsis*. *Plant J.* 45 166–179. 10.1111/j.1365-313X.2005.02585.x 16367962

[B98] Yoshimoto K. Hanaoka H. Sato S. Kato T. Tabata S. Noda T. (2004). Processing of ATG8s, ubiquitin-like proteins, and their deconjugation by ATG4s are essential for plant autophagy. *Plant Cell* 16 2967–2983. 10.1105/tpc.104.025395 15494556 PMC527192

[B99] Yoshimoto K. Jikumaru Y. Kamiya Y. Kusano M. Consonni C. Panstruga R. (2009). Autophagy negatively regulates cell death by controlling NPR1-dependent salicylic acid signaling during senescence and the innate immune response in *Arabidopsis*. *Plant Cell* 21 2914–2927. 10.1105/tpc.109.068635 19773385 PMC2768913

[B100] Yu G. L. Katagiri F. Ausubel F. M. (1993). *Arabidopsis* mutations at the RPS2 locus result in loss of resistance to *Pseudomonas syringae* strains expressing the avirulence gene avrRpt2. *Mol. Plant Microbe Interact.* 6 434–443. 10.1094/MPMI-6-434 8400373

[B101] Yu I. C. Parker J. Bent A. F. (1998). Gene-for-gene disease resistance without the hypersensitive response in *Arabidopsis* dnd1 mutant. *Proc. Natl. Acad. Sci. U.S.A.* 95 7819–7824. 10.1073/pnas.95.13.7819 9636234 PMC22769

[B102] Zhang Y. Goritschnig S. Dong X. Li X. (2003). A gain-of-function mutation in a plant disease resistance gene leads to constitutive activation of downstream signal transduction pathways in suppressor of npr1-1, constitutive 1. *Plant Cell* 15 2636–2646. 10.1105/tpc.015842 14576290 PMC280567

[B103] Zhang Z. Lenk A. Andersson M. X. Gjetting T. Pedersen C. Nielsen M. E. (2008). A lesion-mimic syntaxin double mutant in *Arabidopsis* reveals novel complexity of pathogen defense signaling. *Mol. Plant* 1 510–527. 10.1093/mp/ssn011 19825557

[B104] Zhang Z. Wu Y. Gao M. Zhang J. Kong Q. Liu Y. (2012). Disruption of PAMP-induced MAP kinase cascade by a *Pseudomonas syringae* effector activates plant immunity mediated by the NB-LRR protein SUMM2. *Cell Host Microbe* 11 253–263. 10.1016/j.chom.2012.01.015 22423965

[B105] Zhao T. Rui L. Li J. Nishimura M. T. Vogel J. P. Liu N. (2015). A truncated NLR protein, TIR-NBS2, is required for activated defense responses in the exo70B1 mutant. *PLoS Genet.* 11:e1004945 10.1371/journal.pgen.1004945 PMC4305288 25617755

[B106] Zhou F. Menke F. L. Yoshioka K. Moder W. Shirano Y. Klessig D. F. (2004). High humidity suppresses ssi4-mediated cell death and disease resistance upstream of MAP kinase activation, H2O2 production and defense gene expression. *Plant J.* 39 920–932. 10.1111/j.1365-313X.2004.02180.x 15341634

[B107] Zhou J. Yu J. Q. Chen Z. (2014). The perplexing role of autophagy in plant innate immune responses. *Mol. Plant Pathol.* 15 637–645. 10.1111/mpp.12118 24405524 PMC6638830

